# Detour migration to circumvent the Himalayas in the Montagu’s Harrier *Circus pygargus*

**DOI:** 10.1186/s40462-025-00568-z

**Published:** 2025-06-10

**Authors:** Arjun Kannan, M. B. Prashanth, Abhishek Samrat, Raymond H. G. Klaassen, T. Ganesh

**Affiliations:** 1 Manipal Academy of Higher Education, Manipal, India; 2 SMS Foundation Centre for Biodiversity and Conservation, Ashoka Trust for Research in Ecology and the Environment (ATREE), Royal Enclave, Srirampura, Jakkur Post, Bengaluru, Karnataka 560064 India; 3 Groningen Institute for Evolutionary Life Sciences (GELIFES), University of Groningen, PO Box 11103, 9700 CC Groningen, The Netherlands; 4 Dutch Montagu’s Harrier Foundation, Berkenweg 1, 9471 VA Zuidlaren, The Netherlands

**Keywords:** Migratory bird conservation, Central Asian Flyway, Grasslands, High altitude, Migration patterns

## Abstract

**Background:**

Migrating birds do not always travel along the shortest possible routes between breeding and wintering sites. Rather, detours are a common phenomenon in response to availability of foraging habitats, generic wind patterns, predation risk, and ecological barriers. The Himalayas are a formidable ecological barrier within the Central Asian Flyway (CAF), but hitherto research has focused on high-altitude flights of species that cross the Himalayas, and thus information on species that circumvent this mountain range is lacking in this understudied migration system.

**Methods:**

We tracked Montagu’s Harriers *Circus pygargus* for 5 years from their wintering range in India, and found that these long-distance migrants travelled by a grand westward detour around the Himalayas to their breeding areas in Kazakhstan. We calculate the energetic optimality of the detour on the basis of a well-known theoretical model and explain the general migration patterns of Montagu’s Harriers in the CAF. Additionally, we compare ecological factors such as ground elevation, habitat greenness (NDVI), land cover and wind patterns along the actual migration route with the hypothetical shortest route to explain why Montagu’s Harriers follow a detour migration pattern in the CAF.

**Results:**

The observed (detour) route was on average 1245 ± 94.4 km (27%) longer than the hypothetical shortest direct route. The detour did seem to be optimal for Montagu’s Harriers as per the model that considers a distance of up to 1288 km to be optimal. With the detour, harriers circumvented the high altitudes of the Himalayan plateau, effectively avoiding high ground elevations over 4000 m above mean sea level (AMSL). Harriers followed the same detour during spring and autumn migrations, encountering both supporting and opposing winds, and thus the detour cannot be explained by generic wind patterns. The detour was facilitated by the availability of open natural ecosystems (ONEs) and stopover sites with higher productivity west of the mountain range along the floodplains of the Amu Darya river and in the Thar Desert during spring and autumn respectively.

**Conclusion:**

We argue that circumventing the mountain range, as illustrated by our pioneer study on the Montagu’s Harrier, could be a common behaviour among migrating landbirds in the CAF. We also emphasize the importance of the protecting ONEs along the western detour for the long term conservation of migratory birds in the CAF.

**Supplementary Information:**

The online version contains supplementary material available at 10.1186/s40462-025-00568-z.

## Background

Migrating birds do not always travel along the shortest possible routes between their breeding and wintering sites, but detours are commonly made in response to the availability of refueling habitats [1–3], generic wind patterns [4–6], predation risk [8], and the avoidance of ecological barriers [9, 10]. It is important to understand how detours arise as they have a profound effect on the geometry of bird migration routes, with direct relevance to the conservation of migratory populations [11].

Detours result in longer migration distances, but can nevertheless be optimal when the time and energy required to make the detour (for example to avoid a barrier) is lesser than the time and energy needed to prepare for and undertake the crossing of that barrier [12, 13]. Soaring birds such as raptors may choose a longer detour route to avoid barriers such as sea crossings and mountains where there are limited conditions for undertaking soaring flight, hence resorting to energy demanding flapping flights to undertake the detour [12]. Barriers such as a mountains are also characterized by rapid changes in wind patterns, turbulence and instant cloud formations due to the complex terrain, thus requiring migrants to adapt to the changing conditions which incurs additional energetic costs [14, 15]. Assuming that the transport costs differ between flapping and soaring modes of flight, Alerstam [12] fitted these values to the following equation to calculate the maximal distance (D) up to which the detour can be energetically economical for a bird:

$$\frac{D}{{Y}_{b}} = \left(\frac{{C}_{1}}{{C}_{2}}\right)-1$$

where D is the extra detour distance, Y_b_ is the barrier distance which the bird is expected to cross, C_1_ is the transport costs across the barrier (flapping flight cost) and C_2_ is the transport costs across non-barrier areas (soaring flight cost).

The Himalayas—extending across 72.80°E–97.12°E longitudes to 34.49°N–29.73°N latitudes [16], with an average altitude of 4500 m—form a formidable ecological barrier within the Central Asian Flyway (CAF) [17]. Research on migratory birds in this region has focussed on species that cross the high altitudes of the Himalayan plateau, such as the Bar-headed Geese *Anser indicus* [18], Ruddy Shelduck *Tadorna ferruginea* [19], Demoiselle Crane *Grus virgo* [20] and Black-eared Kite *Milvus migrans* [21], and the special physiological adaptations related to high-altitude migration [17, 22]. Tracking studies on songbirds such as the Siberian Rubythroat *Calliope calliope* in the CAF have shown that they exhibit differential migration strategies with individuals detouring to avoid high elevations of the Himalayas in autumn, but crossing over mountainous areas in spring to follow a more direct migration route [23]. A handful of tracking studies on raptors have highlighted the importance of circum-Himalayan corridors in the CAF especially for species such as the Pallid Harrier *Circus macrourus* which is known to migrate via the western-circum Himalayan corridor [24] and the Amur Falcon *Falco amurensis* that migrates via the eastern-circum Himalayan corridor [25]. Thus, these species clearly seem to be avoiding the high altitudes of the Himalayan plateau. Meanwhile, satellite tracking of Peregrine Falcons *Falco peregrinus* have shown that they cross the Himalayas on a broad front with many individuals overwintering at altitudes of > 4500 AMSL on the Tibetan Plateau [26]. This overwintering strategy has also been exhibited by Saker Falcons *Falco cherrug* (Potapov et al., [27]) and Steppe Eagles *Aquila nipalensis* [28], although only one individual of each species was tracked. However, detailed knowledge of how the high altitudes of the Himalayas acts as a barrier for migratory birds, and how migrating birds, especially raptors strategize to either cross over or avoid this mountainous barrier in the CAF remains to be understood [29]. This understanding can only be achieved with data from high resolution tracking of migrant bird species in the CAF.

In this study, we provide data on the detour migration of the Montagu’s Harrier *Circus pygargus* circumventing the Himalayas during their migration between the breeding sites in Kazakhstan and wintering sites on the Indian subcontinent. This medium-sized raptor restricted to open natural ecosystems (ONEs) such as arid and semi-arid grasslands [30] is a species of conservation concern as their populations have been under consistent decline across India in the past decades for reasons unknown [31, 32]. Montagu’s Harriers travel by a combination of flapping and soaring flight [33], which makes them relatively flexible in their responses to variations in atmospheric conditions [34]. Furthermore, they are able to adopt a fly-and-forage migration strategy combining foraging and travelling [35], taking advantage of favourable foraging conditions along their migration routes.

We quantify the extent of the detour Montagu’s Harriers make during spring and autumn migration, and investigate the possible ecological drivers that shape their routes. Additionally, we test if the detour migration undertaken by Montagu’s Harriers is optimal based on Alerstam’s [12] model. We hypothesize that Montagu’s Harriers make the detour to circumvent the high altitudes of the Himalayan plateau. To test this idea, we compare the ground altitudes the birds encountered along their detour routes with the ground altitudes along a hypothetical direct route crossing the Himalayas. We subsequently explore to what extent the detour route is supported by favourable foraging conditions, in particular the distribution of ONEs, by characterising the habitats along the detour route and the hypothetical direct route. We use habitat greenness (NDVI) as a proxy for productivity at the major stopover sites used by Montagu’s Harriers during migration. Finally, we explore to what extent the detour route is supported by generic wind patterns by performing a wind drift analysis to quantify the (tail) wind support between the detour and the hypothetical direct route and by quantifying how Montagu’s Harriers respond to crosswinds along the detour migration route.

Our detailed description of the detour migration of Montagu’s Harriers around the Himalayas in relation to ground altitude, habitat cover, NDVI at stopover sites and wind patterns, provides novel insights into the behaviour of migratory birds within the CAF.

## Methods

The datasets used and subsequent analyses performed using each of the datasets have been provided in the sections below. All analyses were conducted in R statistical software version 4.3.1 [36] and all graphs were plotted using the ggplot2 package (Wickham [37]) within R.

### Tracking data

Six Montagu’s Harriers (4 males and 2 females) were tagged using 9.5 g Platform Telemetry Terminals, from here on, PTT tags (PTT-100, Microwave Telemetry Inc., Colombia, USA) and four Montagu’s Harriers (1 male and 3 females) were tagged with 9.5 g GPS-GSM tags (GsmTag-U9*,* Milsar technologies, Sat Gheorghieni, Romania). The tags were fitted onto the backs of the individuals using a 5.6 mm Teflon full body harness (Bally Ribbon Mills, Bally, PA, USA). The PTT tags were only providing location data (see details below) whereas the GPS-GSM tags also provided altitude data (above mean sea level) along with each location.

The PTT tags were programmed with a duty cycle of 10 h ON (transmitter is sending data) and 48 h OFF (transmitter is not sending data). The number of location fixes during “on-cycles” depends on the configuration and passing of satellites receiving the transmitter signals. During each transmission cycle, a number of location points of variable quality (indicated as ARGOS location error estimate ‘location class’, LC) was obtained. We used LC 1, 2 and 3 as these were the most accurate locations (< 150 m, accuracy according to the manufacturer). LC 0 class locations were used only if the points did not show a significant deviation from the migration route of the birds cf. Limiñana et al. [38]. LC A or B location class locations were not used as they were deemed too inaccurate. The GPS-GSM tags were programmed to record locations at a 10-min interval during the day (from 00:00 UTC to 14:00 UTC) and at 1 h interval during the night (from 14:01 UTC to 23:59 UTC). Accuracy of the positions obtained by the GPS-GSM tags when recording locations at 10 min intervals is typically within 30 m [39].

Harriers were tagged in the years 2016–2020 at their wintering sites in India at Tal Chhapar Sanctuary (27.81°N, 74.43°E), fringe areas of the Nannaj Bustard Sanctuary (17.82°N, 75.86°E) and grazing lands in Tirunelveli, Tamil Nadu State (8.54° N, 77.65° E). Individuals were captured in the evening at their night roosts using a 12 m mist net and a decoy of an Indian Eagle Owl. Harriers were trapped when mobbing the decoy owl. After capture, morphometric measurements of the individuals were taken before fitting a metal or a plastic leg ring and a PTT or a GPS-GSM tag. We avoided tagging birds which were moulting or had any signs of injuries. Since the birds were captured late in the evening, it was not possible to release them in the night as they might be disoriented and can be predated. Instead, the birds were kept overnight in a well aerated cardboard box with a layer of grass bedding and were released during the early hours of the following day at the site of capture. One of the PTT tags (Montagu’s Harrier female) stopped functioning before the onset of the first migration (2018) and this individual has been excluded from all analyses. Thus, the total dataset consisted of 40 migration tracks of 9 individuals (Table S1).

The tracking dataset consisted of 17,551 location points (15,370 GPS and 2,181 PTT locations). From the dataset, migration tracks were extracted by plotting the tracks on Google Earth Pro (version 7.3.6976) for manually determining when the birds left their last wintering site in India (for spring migration) or breeding site (for autumn migration), and when the breeding site (for spring migration) or first wintering site in India (for autumn migration) was reached. Stopover sites during migration were defined as sites where harriers paused their migratory journeys, i.e., travelled less than 50 km/day [40] for more than 3 days. Although harriers did make several short stopovers (< 3 days) during migration, we considered only the longer true stopovers (> 3 days) for the analysis. All birds completed at least one migration cycle (autumn and spring). The maximum number of migration cycles for an individual was 5. Since the tracking dataset consisted of information from only 9 tagged individuals, all subsequent analyses were performed at the level of each migration track and not at the level of individual harriers.

For each Actual Migration Route (AMR), the corresponding Great Circle Route (GCR) was determined between the start and end point of the migration route. The GCR represents the shortest possible direct route. Coordinates at every 100 km along the GCR were extracted using the *gcIntermediate* () function in the Geosphere package version 1.5-20 [41]. A total of 1433 location points were extracted along the GCR. The AMR and GCR distances for each migration track were calculated with the *distGeo* () function within the Geosphere package. For the AMR, each migration track was individually plotted on Google Earth Pro (version 7.3.6976) and all stopover points were filtered out. For calculating travel distance and travel speeds, only migrating movement points were considered in order to minimise the bias of movements within a site (stopover) inflating the total migration travel distance. After calculating the travel distance for each migration track, the travel speed was calculated by dividing the total number of days to complete the migration (including days at stopover) with the travel distance. We also calculated the active travel speed by dividing the number of travelling days (excluding days at stopover) by the travel distance. In order to see if there was any difference in travel speeds recorded between the PTT and the GPS-GSM tags, a t-test was performed using the *t.test* () function. The relative extent of the detour was calculated for each spring and autumn migration track by dividing the difference in the length of the AMR and the GCR by the length of the GCR cf. Vansteelant et al. [7].

### Calculating optimality of the detour

The transport costs for flapping and soaring flights were calculated based on Pennycuick’s [42] aerodynamic theory of bird flight. The transport cost of flapping flight was calculated as:

$${C}_{1} = \frac{{P}_{mr}}{{V}_{mr}}$$

where P_mr_ is the power at maximum range speed and V_mr_ is the maximum range speed attained the bird during migration. The transport cost of soaring flight was calculated by:

$${C}_{2} = \frac{{P}_{s}}{{V}_{cc}}$$

where P_s_ is the power required for soaring flight and V_cc_ is the maximum cross country speed which can be attained by the bird. Since we do not have real time measurements of speed and the power requirements of Montagu’s Harriers, we used the afpt package version 1.1.0.4 in R [43] which models animal flight performance by plugging in morphological measurements such as mass of the bird, wingspan and the wing area [42]. The morphometric measurements for the Montagu’s Harrier were taken as: mass = 0.291 kg, wingspan = 1.09 m and wing area = 0.135 m^2^; which were based on the measurements provided by Alerstam [44]. These mass and wingspan measurements were very close to our own measurements from the captured Montagu’s Harriers. The P_mr_, V_mr_ and V_cc_ values were computed using the *computeFlightPerformance* () function in the afpt package in R, while the P_s_ was calculated as 4 times the Basic Metabolic Rate (BMR) of the bird (cf. [45]). The BMR for the Montagu’s Harrier was calculated using the equation provided by Laseiwski and Dawson [46].

To calculate the maximum detour distance which can be energetically optimal for the bird we used Alerstam’s [12] equation where the barrier distance, Y_b_ for the Himalayas was calculated by first plotting all the GCR tracks over a digital elevation model (see next section) on Quantum GIS (version 3.28.10) and the measure tool was used to measure the distance of each track which crossed (N = 40) over the Himalayas at altitudes of > 3500 m (AMSL), as majority of the location points (~ 99%) recorded by the GPS-GSM tagged birds were below this altitude. The Y_b_ was then calculated as 700 km, which roughly is the average distance of each of these tracks crossing over the Himalayas.

### Ground elevation data

Ground elevation data along the AMR and the GCR was obtained from the USGS Shuttle Radar Topography Mission, Digital Elevation Model, 90 × 90 m resolution (Consortium for Spatial Information (CGIAR-CSI), https://srtm.csi.cgiar.org/). Positions along the AMR and GCR were overlaid on the digital elevation model and the corresponding elevation was extracted using the Point Sampling Tool plugin in Quantum GIS (version 3.28.10). For birds with GPS tags (N = 4), we also calculated altitudes above the ground by subtracting the ground elevation for each location point from the altitude recorded by the tags. To test for differences in ground altitudes between AMR vs GCR and between the autumn and spring migration seasons, a Wilcoxon signed-rank test was performed with the *wilcox.test* () function in R. Altitudinal differences across latitudes were also tested using the Wilcoxon signed-rank test by grouping the location points at 10° latitude intervals (5 categories: 8°N–18°N, 18°N–28°N, 28°N–38°N, 38°N–48°N & 48°N–58°N). The relationship between absolute flight altitudes above mean sea level (AMSL) recorded by the GPS-GSM tagged individuals and ground elevation was tested using a linear regression model with the *lmer* () function in the lmerTest package [47] in R. A similar linear regression was also run to test the relation between the flight altitudes above ground (difference between actual altitude recorded by the GPS-GSM tags and the ground elevation at that location point) and ground elevation. The residuals of the models were checked for normality with the Shapiro–Wilk test with the *shapiro.test* () function and the R^2^ values were computed to check the goodness of fit.

### Habitat greenness data (NDVI)

Seasonality in the habitat productivity at the main stopover sites (sites used for a duration of more than 3 days) was studied on the basis of the Normalized Difference Vegetation Index (NDVI), which measures plant greenness and vegetation density [48]. Main stopovers were made in two areas, as used by multiple individuals across multiple years. For the two areas a 100% Minimum Convex Polygon (MCP) of the recorded location points from the birds was drawn over these main stopover locations in Quantum GIS (version 3.28.10). Subsequently, monthly means of NDVI values were then extracted for these MCPs (2 sites) for all months from the Terra Moderate Resolution Imaging Spectroradiometer (MODIS) MOD13Q1 V6 [49] NDVI product. MOD13Q1 V6 has a 16-day temporal resolution and 250 m spatial resolution. The pre-processed MOD13Q1 was downloaded using the cloud computing platform Google Earth Engine [50]. A times series of the mean NDVI for each month was then plotted to visualise how productivity changed across the months.

### Land cover data

Land cover data along the AMR and the GCR was obtained for the year 2019 from the European Space Agency Land Cover Climate Change Initiative (ESA Land Cover CCI) dataset [51]. This yearly dataset has a 300 m spatial resolution. The dataset has a total of 37 Land Cover Land Use (LCLU) thematic classes. Since many of the thematic LCLU categories had minor ecological differences between them (such as sparse vegetation and sparse shrub) we reduced the LCLU categories to 8 main thematic classes relevant to the ecology of Montagu’s Harriers namely: Cropland, Cropland mosaic, Forest, Open natural ecosystem (ONE), Urban areas, Bare areas, Water body, and Permanent snow and ice (Table S2). Location points along the AMR and GCR were overlaid on the ESA Land Cover map and the corresponding land cover class was extracted using the Point Sampling Tool Plugin in Quantum GIS (version 3.28.10). Two proportion Z-tests were performed using the *prop.test* () function in R to test for differences between the proportions of location points falling on each land cover class between AMR and GCR and also between the autumn and the spring migration seasons.

### Wind data

Location data from the PTT tags were divided into movement segments of > 50 km of at least 4 h, indicating migratory flights. Only daytime positions were considered as Montagu’s Harriers are relatively strict diurnal migrants [35]. For the more accurate GPS tracks, daily segments which includes the first and last position of each day were extracted. The complete dataset consisted of 338 movement segments. Subsequently, wind data was obtained from the NCEP Reanalysis II dataset, using the RNCEP package [52] in R. NCEP data consists of east–west (u-wind) and north–south (v-wind) components (expressed in m/s), at a spatial resolution of 2.5° × 2.5° and a temporal resolution of 6 h (being available at 00:00, 06:00, 12:00, and 18:00 h UTC). Following Klaassen et al. [53] and Mellone et al. [54] wind data was extracted for a pressure level of 925 hPa, which corresponds to an altitude of about 750 m. For each segment, wind components were extracted for the start, middle and end point, interpolating in space and time (using the *NCEP.interp* () command in RNCEP). To simulate the effect of wind experienced along a migration segment, the average wind component was calculated, giving more importance to the wind at the midpoint of the segment (cf. [53, 55]) by:

$${u}_{ wind \,segment }=\frac{\left( {u }_{wind \,onset }+2*{u }_{wind\, midpoint}+ {u}_{ wind\, end}\right)}{4}$$

$${v}_{ wind \,segment }=\frac{\left( {v }_{wind\, onset }+2*{v }_{wind \,midpoint}+ {v}_{ wind\, end}\right)}{4}$$

Finally, from the average u- and v-wind components, the wind direction (in degrees) and wind velocity (in m/s) were calculated.

A wind drift analysis was conducted following Thorup et al. [56], using the direction between the start and end point of the whole migration track as the intended or preferred direction of movement. Subsequently, we calculated the forward (tailwind) and perpendicular (crosswind) components of the wind (in m/s) in relation to this intended direction, as well as the forward and perpendicular movement components (in km/day) [53, 56]. The degree of perpendicular drift and the forward movement was quantified by performing a linear regression model using the *lm* () function in R between perpendicular movement component (response variable) and crosswind, and the correlation between forward movement component (response variable) and tailwind respectively. Finally, we classified, for every movement segment, whether the bird had been drifting, compensating or overcompensating, following the approach by Klaassen et al. [53]. Drift is defined as a perpendicular movement of more than 50 km/day or less than − 50 km/day, with similar signs for perpendicular movement and perpendicular winds. Perpendicular movement corresponding to less than 50 km/day and more than − 50 km/day was classified as compensation. Overcompensation is a perpendicular movement of more than 50 km/day or less than 50 km/day with opposite signs for the perpendicular movement and perpendicular wind components. The frequencies of drift, compensation and overcompensation segments were summarised across three latitudinal bands (< 30°N, between 30°N and 40°N and > 40°N) along the migration routes of the birds. The central latitudinal band is the area where the Montagu’s Harriers circumvent the Himalayan plateau. In order to test if the frequencies of drift behaviour varied between latitudes and between the migration seasons, chi-square tests were performed.

In order to quantify the difference in tailwind support along the AMR compared to the GCR, tailwind components were also extracted for the GCRs. To be able to do this, the AMR locations were projected on the GCR by replacing the longitudes of the AMR with the corresponding longitudes of the GCR thus allowing us to use the same timestamp for each of the segments. Differences in the distribution of tailwinds for the AMR and the GCR were tested for a total of 307 movement segments with t-test (using the *t.test* () function in R).

## Results

### Migration patterns and optimality of the detour

**Fig. 1:**
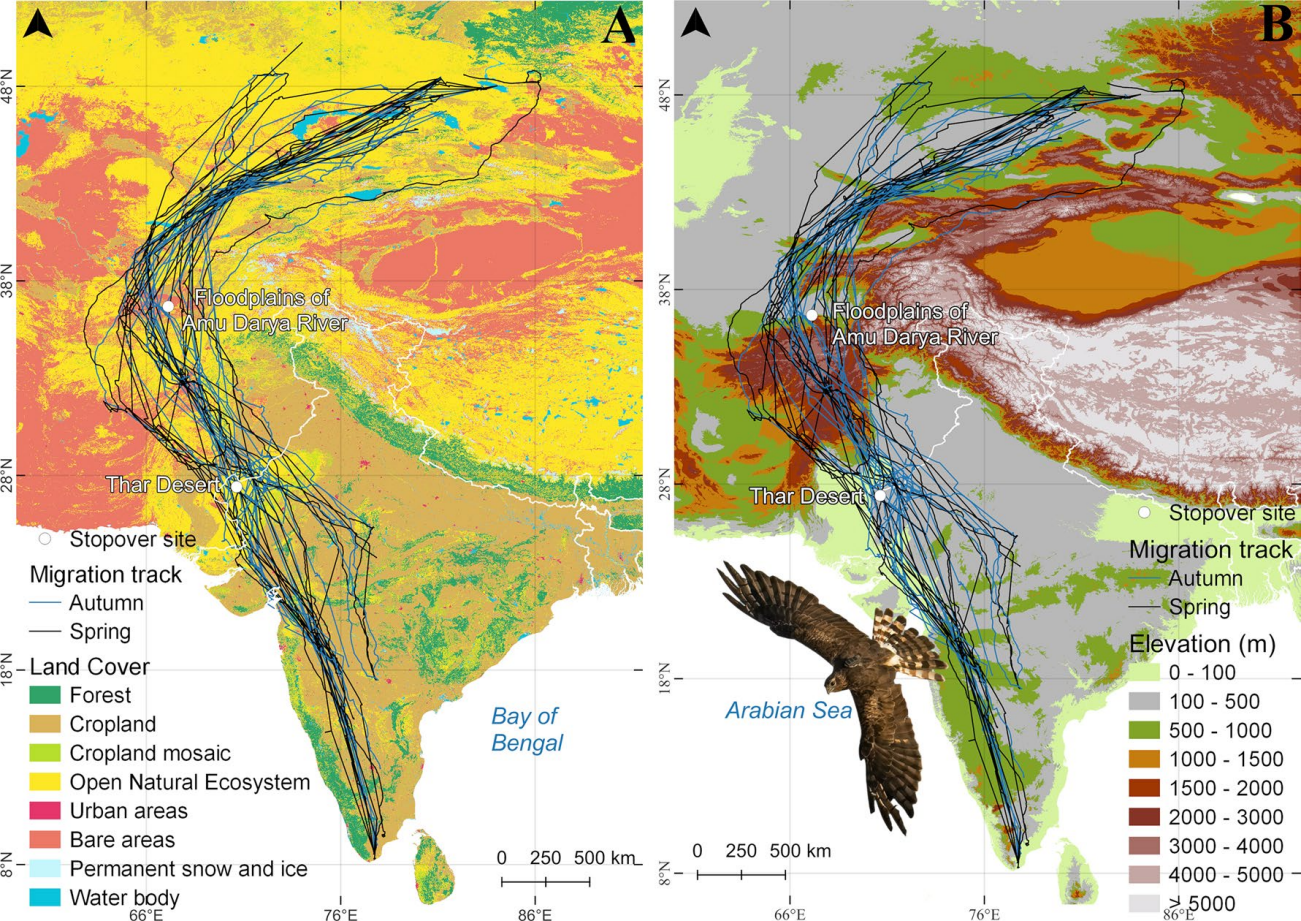
**a** Spring (Black) and autumn (Blue) migration routes of Montagu’s Harriers *Circus pygargus* tracked during the years 2017–2021 overlaid on a land cover map and **b** a digital elevation map. 40 migration tracks have been presented on these maps. The two white dots on the map represents the two major stopover sites of Montagu’s Harriers. Thar Desert is mainly used as a stopover during autumn migration while the floodplains of the Amu Darya river is used as a spring stopover site (The harrier graphics in the figure was prepared using a photograph captured by Mr. Vipul Ramanuj)

The Montagu’s Harriers tagged at their wintering sites in India migrated via a western detour, circumventing the Himalayas but crossing over the western stretches of the Hindu Kush range, to their breeding sites in Kazakhstan (Fig. 1a and b). The tags provided data for 20 autumn and 20 spring migration tracks of 9 individuals (Table S1; Table 1). Out of the 40 tracks, 30 were from PTT tags and 10 were from the GPS-GSM tags. Autumn migration routes were similar to the spring routes. The detour route taken by harriers was on average 1245 ± 94.3 kms (27% mean ranging from 11 to 58% for individual tracks) longer than the great circle route.

**Table 1:** Mean estimates of migration distance and time during spring and autumn by Montagu’s Harriers from 2017 – 2021

Migration	N^*^	Spring migration Mean ± SE (range)	N^*^	Autumn migration Mean ± SE (range)	t	*P*
Departure date^#^	20 (9)	88.1 ± 1.86 (80–113)	20 (9)	227.5 ± 3.89 (181–248)		
Arrival date^#^	20 (9)	113.84 ± 1.89 (104–137)	20 (9)	273.87 ± 4.84 (253–317)		
Total duration (days)	20 (9)	26.36 ± 0.97 (19–33)	20 (9)	45.52 ± 5.73 (11–98)	299.5	**0.007**
No. of traveling days	20 (9)	22.8 ± 1.12 (12–31)	20 (9)	22.05 ± 1.80 (11–36)	−0.35	0.7
Distance traveled (km)	20 (9)	4832.24 ± 207.63 (3270.35–5938.25)	20 (9)	4510.69 ± 242.41 (2678.35–6502.43)	−1.009	0.3
Great circle route (km)	20 (9)	3571.32 ± 154.36 (2442.09–4534.72)	20 (9)	3280.78 ± 169.1 (1981.11–4393.81)	164	0.3
Travel speed (km/day)	20 (9)	185.03 ± 7.01 (131.38–236.87)	20 (9)	122.81 ± 13.16 (53.92–315.71)	−4.17	** < 0.01**
Active travel speed (km/traveling day)	20 (9)	217.64 ± 10.44 (150.96–357.85)	20 (9)	216.80 ± 10.71 (160.84–315.71)	−0.05	0.95
No. of stopovers	20 (9)	0.78 ± 0.24 (0–4)	20 (9)	1.94 ± 0.31 (0–6)	317.5	**0.001**
Days at stopover		3.88 ± 1.04 (0–15)		23.45 ± 4.84 (0–69)	362	** < 0.01**

Significant *p*-values have been highlighted in bold

^*^Number of migration tracks (number of tagged individuals)

^#^Julian date (1 = 1 January)

Breeding sites were located in east (catchments of Lakes Balkhash, Alakol and Zysan) and central Kazakhstan. The land cover in this region is primarily dominated by steppes (saline and dry) and semi-desert plains with hills, and grasses interspersed with agricultural lands (Sánchez-Zapata et al., [57]). Two individuals occupied sites in central Kazakhstan which mainly consisted of open grasslands interspersed with farmlands (as determined from Google Earth Pro imagery version 7.3.6.9796). Non-breeding (wintering) ranges extended from western to southern India. Here the harriers occupied arid and semi-arid grasslands interspersed with agricultural lands.

The main stopover site during autumn migration was in the Thar Desert region (Fig. 1a) bordering India and Pakistan (28.16°N, 71.47°E). It was used by all nine individuals in nineteen out of twenty autumn migration instances. This stopover site was also used during spring migration, but only thrice by three individuals for a short duration (< 3 days). During spring migration, the floodplains of the Amu Darya river (37.11°N, 66.85°E) in Afghanistan was used as a stopover site, but only in 4 out of 20 occasions. There were other short spring stopovers (five sites) but all these sites were used only once by the migrating individuals and for short durations (< 3 days).

The distance covered during migration did not differ between autumn (4510 ± 242 kms) (mean ± standard error) and spring (4832 ± 208 km) (Table 1) migration. However, the duration of migration was on average longer in autumn (45.5 ± 5.7 days) than in spring (26.4 ± 0.97 days) (Table 1). This was due to a difference in the number of stopover days (23.5 ± 4.8 days in autumn versus 3.9 ± 1.0 days in spring) rather than the number of travel days (22 ± 1.8 days in autumn versus 22.8 ± 1.1 days in spring). However, the travel speed was significantly higher in spring (185.0 ± 7.0 km/day) compared to autumn (122.8 ± 13.2 km/day) migration. There was no significant difference between the travel speeds recorded by the PTT and GPS-GSM tags (t = 1.24, *p* = 0.11).

The transport costs for flapping flight (C_1_) was calculated to be 1.16 joules (J). The transport cost for soaring flight (C_2_) was calculated to be 0.408 J. Thus, the ratio between C_1_/C_2_ was 2.84. Assuming a barrier distance Y_b_ of 700 km, Alerstam’s [12] equation gives a maximum detour distance of 1288 km. This is larger than the detour distance (1245 ± 94.3 km) flown by Montagu’s Harriers to undertake the detour migration.

### Ground elevations and flight altitudes

**Fig. 2:**
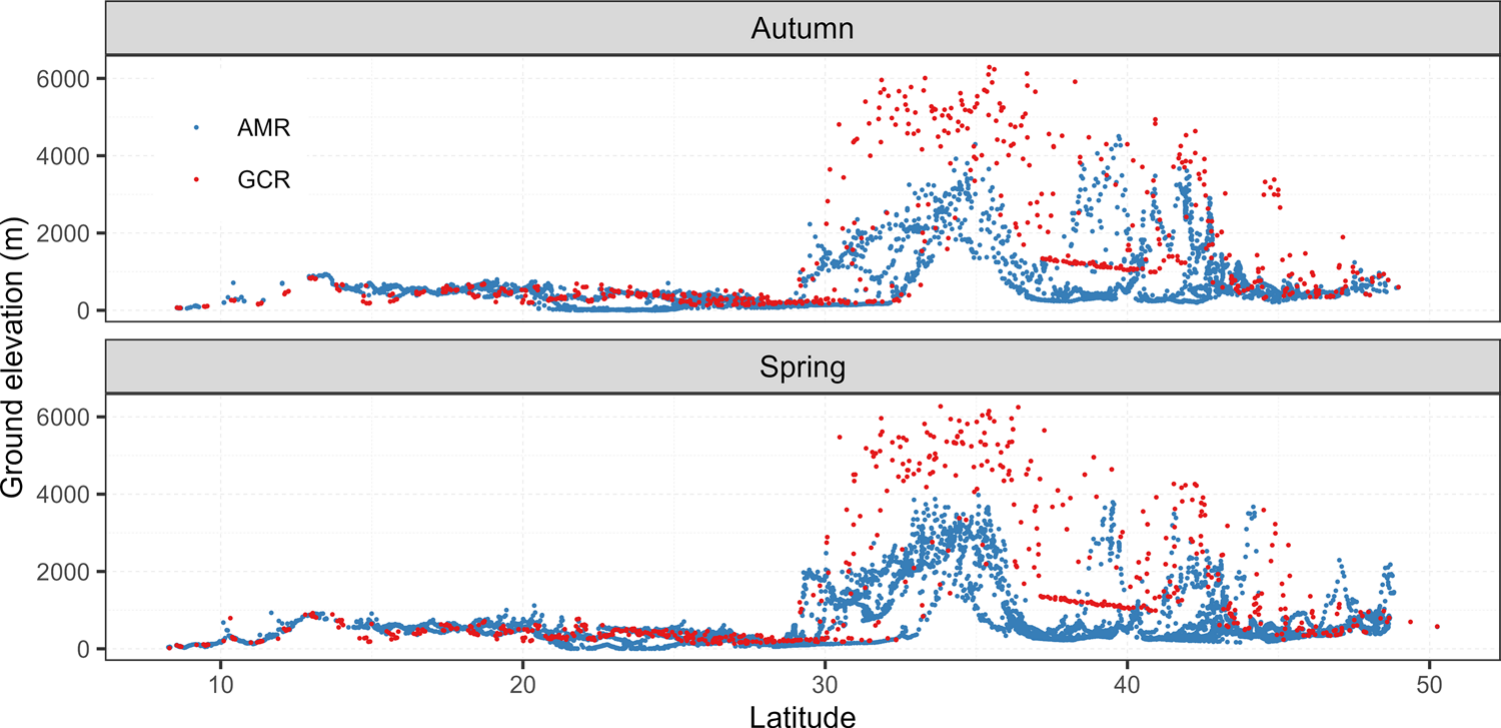
Figure showing the ground elevations (x-axis) along the Actual Migration Route (blue scatter) of Montagu’s Harriers and the hypothetical Great Circle Route (red scatter) across latitudes (y-axis) (N = 9 individuals)

Although the migratory routes (AMR) of Montagu’s Harriers did not statistically differ between autumn and spring, there was a significant difference in the ground elevation profiles between the two seasons (Figs. 1b and 2; Table S3). The mean elevations the harriers crossed during autumn migration was 614 ± 7.06 m, while for the spring migration it was 719 ± 7.25 m. On the contrary, there was no significant difference between ground elevation profiles for the GCR in autumn (1543 ± 66.68 m) and spring (1502 ± 61 m).

Looking at the latitudinal bands, south of the Himalayas (latitude < 28°N), ground elevations did not exceed 1000 m, for both the AMRs and the GCRs (average ground elevation in both cases < 600 m). High ground elevations (> 1000 m) occurred north of > 28°N. Harriers crossed over relatively low mountain ranges, and the ground elevations along their tracks (AMR) were seldom higher than 3500 m (only 46 out of 17,551 locations, < 1%). In comparison, along the GCRs, ground elevations regularly exceeded 3500 m (255 out 1433 locations, 17.7%).

The largest difference in ground elevations between the AMRs and the GCRs was found for the latitudinal band of 28°N to 38°N. Here, the average ground elevation was 927 ± 793 m for the AMRs (with a maximum of 4503 m) and 2358 ± 1827.2 m for the GCRs (with a maximum of 6291 m) (Figs. 1b and 2; Table S3). This difference was significant (Table S3). A similar difference in ground elevations was also noted for the 38°N to 48°N latitudinal band with the AMR having lower elevations of 739 ± 8.1 m compared to the GCR with 1609 ± 58.2 m. Ground elevations for the most southern latitudinal band (8° to 18°N) were also significantly different between AMR and GCR, although the average elevations were low, 509 ± 5.1 m and 410 ± 18.6 m respectively. The most northern latitudinal band (48°N to 58°N) also had low ground elevations for both the AMRs and the GCRs, with average elevations of 745 ± 14.3 m and 760 ± 39.4 m, respectively, but these differences were not significant (Figs. 1b and 2; Table S3).

**Fig. 3:**
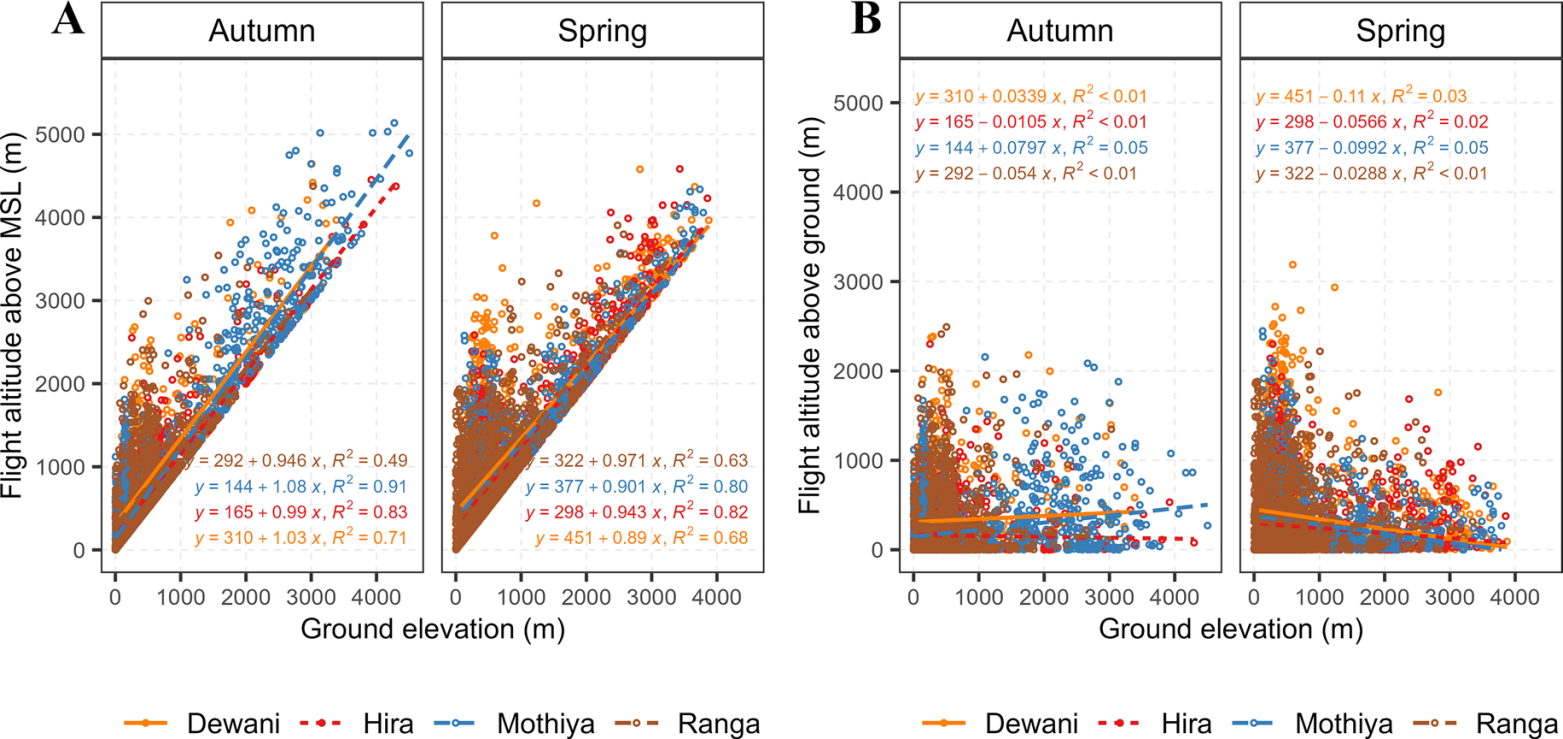
**a** Flight altitudes of Montagu's Harriers above mean sea level (y-axis) recorded by GPS-GSM tags and ground elevation (x-axis) in autumn and spring migrations (N = 4 individuals). **b** Graph depicting flight altitudes above the ground (y-axis) and ground elevation (x-axis) in autumn and spring. Both graphs show the actual migration routes of harriers. The legend shows the IDs of the tagged harriers and the results of the linear regression models have been presented on the graph accordingly

The 4 individuals equipped with GPS-GSM tags provided data on flight altitudes of the birds (Fig. 3a), including the altitudes they flew above the ground (Fig. 3b). The flight altitude above the ground was on average 277.3 ± 4.1 m, this did not differ with the ground elevation (i.e. no relationship between altitude above the ground and ground elevation) (Fig. 3b). Generally, harriers rarely flew above 4000 m AMSL (only 51 out of 15,370 GPS-GSM locations, <1%) (Fig. 3a and b).

### Habitat and resource along the migration route

**Fig. 4:**
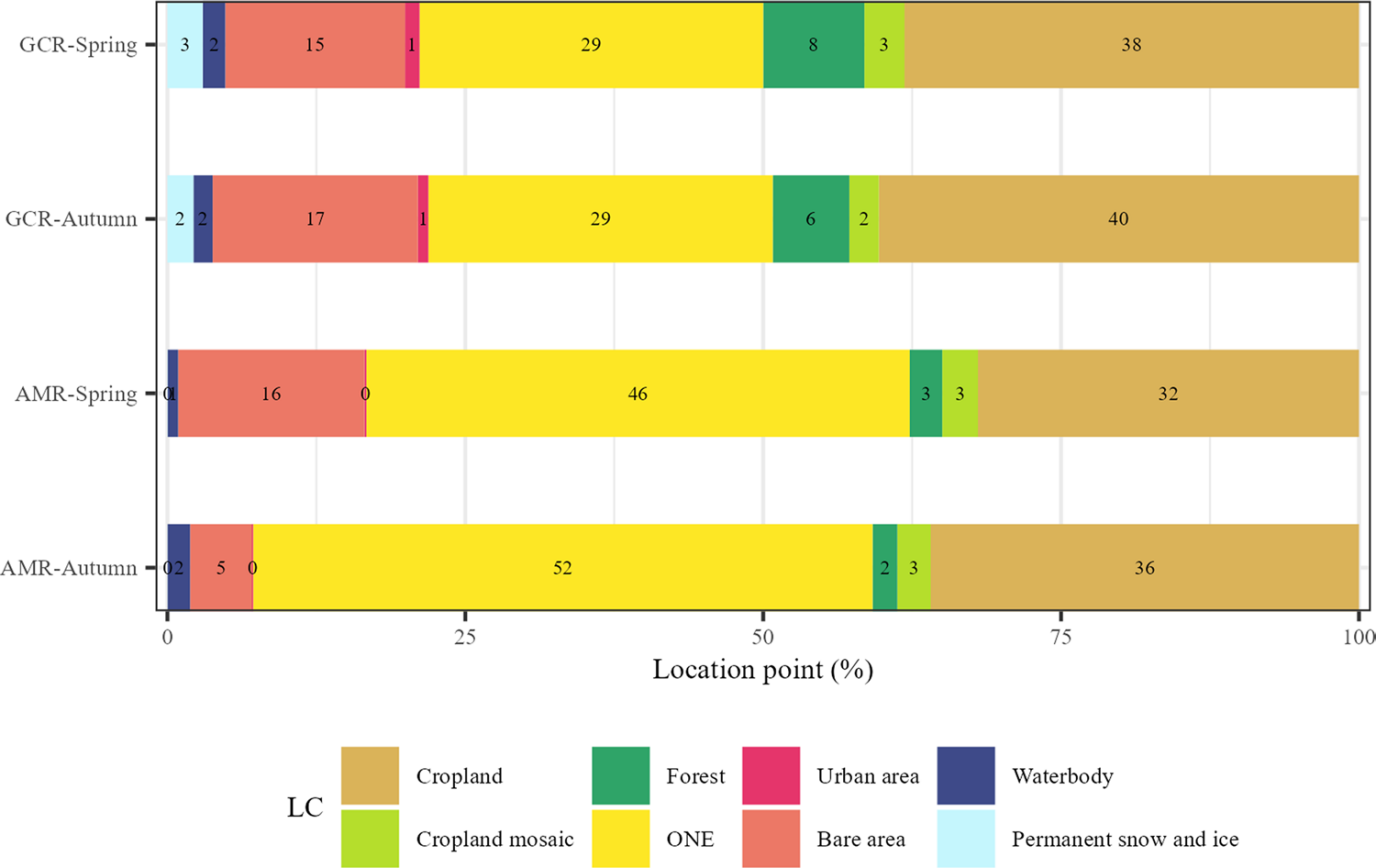
Percentage of recorded location points on different land cover types along the actual migration route (AMR) and the great circle route (GCR). Both spring and autumn AMR and GCR are shown separately

Land cover along the migration route is generally dominated by “Cropland” and “Open Natural Ecosystems” (Fig. 1a). Along the AMR, “Open Natural Ecosystems” was more dominant, whereas “Cropland” dominated the GCR (Fig. 4; Table S4). “Forests” were more abundant along the GCR compared to the AMR. “Permanent snow and ice” was exclusively found only along the GCR. Land cover differences between the seasons were small, although “Bare areas” were more abundant along the AMR in spring than in autumn.

**Fig. 5:**
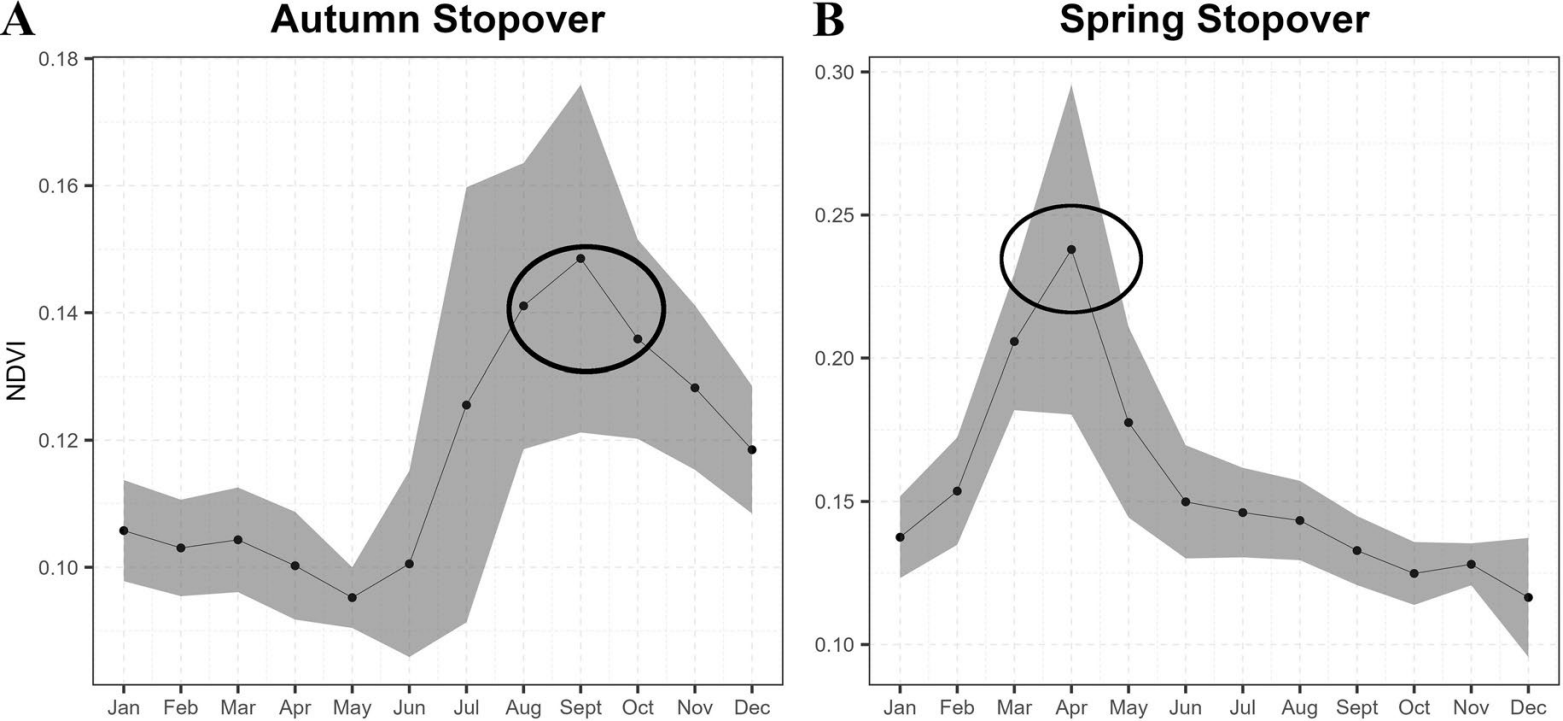
Figures showing the NDVI across months at the autumn stopover at Thar Desert and the spring stopover at the floodplains of the Amu Darya river. The values show the mean NDVI for each month for the years 2017–2021. The grey shaded areas represent the confidence intervals. The ellipses on the figures depict the month(s) at which Montagu's Harriers used these regions as stopover sites

Montagu’s Harriers timed their stopovers during peak habitat greenness/productivity (NDVI) at both the autumn and spring stopover sites (Fig. 5). The highest NDVI for the autumn stopover site was 0.15 in September while for the spring stopover site, it was 0.26 in March and April.

### Movements in relation to wind

**Fig. 6:**
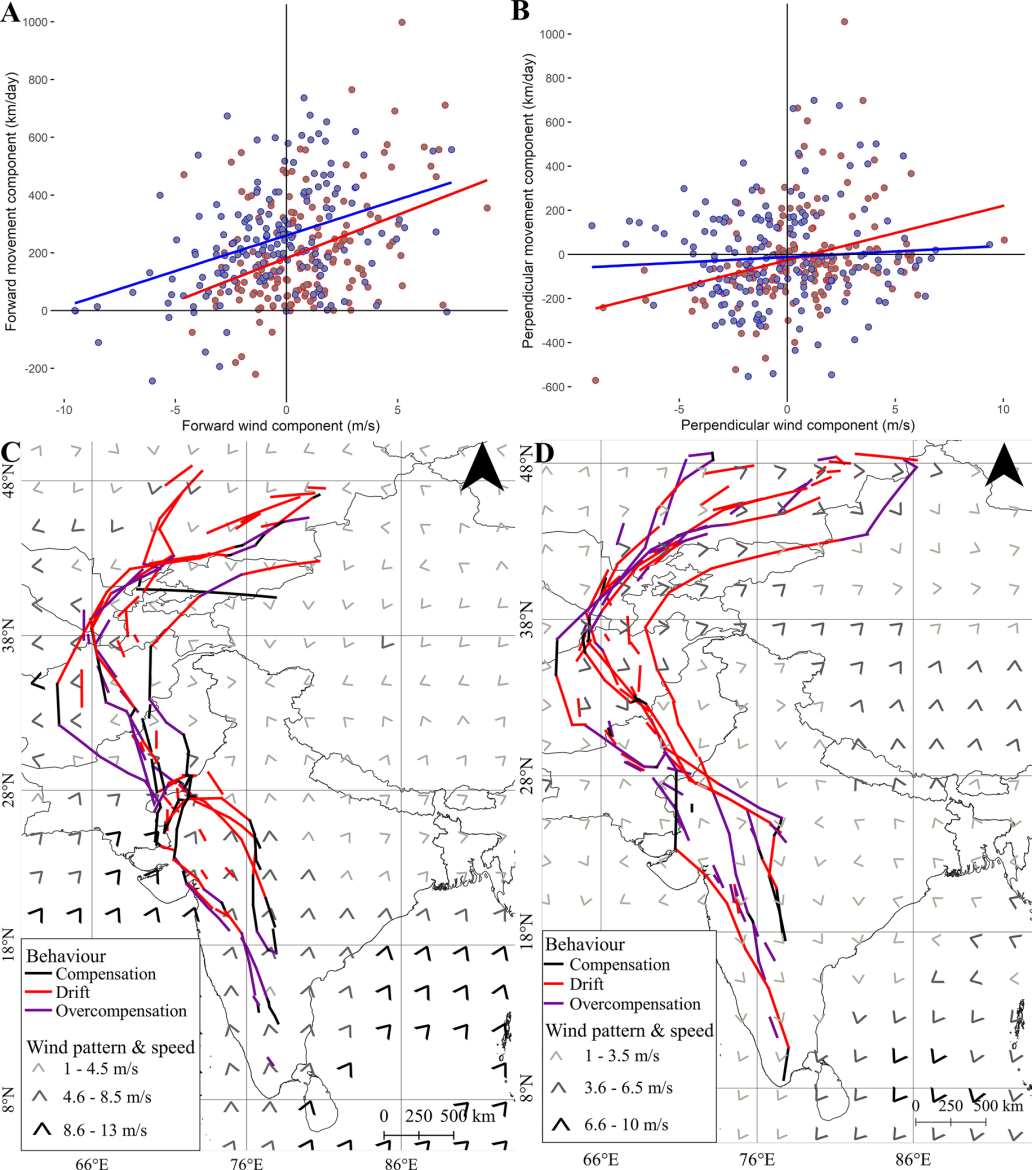
**a** Relationship between forward movement and the forward wind component. **b** Relationship between perpendicular movement and perpendicular wind component. The blue scatters depicts spring migration while the red scatters depict autumn migration. **c** Average wind direction and wind speed during the years 2017–2021 in the CAF overlaid with segments showing behaviour of harriers in the presence of crosswinds for the autumn migration season (N = 169 segments) and the **d** spring migration season (N = 169 segments)

Movements of Montagu’s Harriers were significantly affected by tailwinds (Fig. 6a, Table S5) with their rate of forward movements increasing by 23.2 km/day for every extra meter per second of tailwind (Table S5). The effect of crosswinds on perpendicular movement differed between the seasons (Fig. 6b, Table S5). In autumn, the effect of crosswinds on perpendicular movement was significant, with the latter increasing by 24.7 km/day for every meter per second of crosswind. In spring, the increase in perpendicular movement was only 5.1 km/day for every meter per second of crosswind. This correlation was not significant. Overall, harriers seem to generally drift in autumn and compensate in spring.

In autumn, the Montagu’s Harriers regularly encountered supportive northeastern winds during their initial south-westward movements (Fig. 6c; Table S6). Consequently, we observed a high proportion of drift at latitudes > 40°N. At intermediate latitudes (between 30°N and 40°N), birds often experienced easterlies while moving southwards, resulting in a high proportion of compensation and overcompensation. i.e., for this part of the travel the winds were often not supportive. For the last migration leg, south of 30°N, winds were blowing from the southwest. The birds allowed quite an extent of drift, resulting in a (south) eastern movement, but most of the time winds were opposing in this region (i.e. relatively strong headwind component).

In spring, generic winds were variable at the initial migration leg (south of 30°N), and thus we saw a combination of drift and overcompensation (Fig. 6d; Table S6). At intermediate latitudes (between 30° and 40°N), the birds entered the northern band of supportive western winds. Here we saw a larger component of drift segments. At the last migration leg, western winds still dominated, which resulted in a combination of drift and overcompensation.

**Fig. 7:**
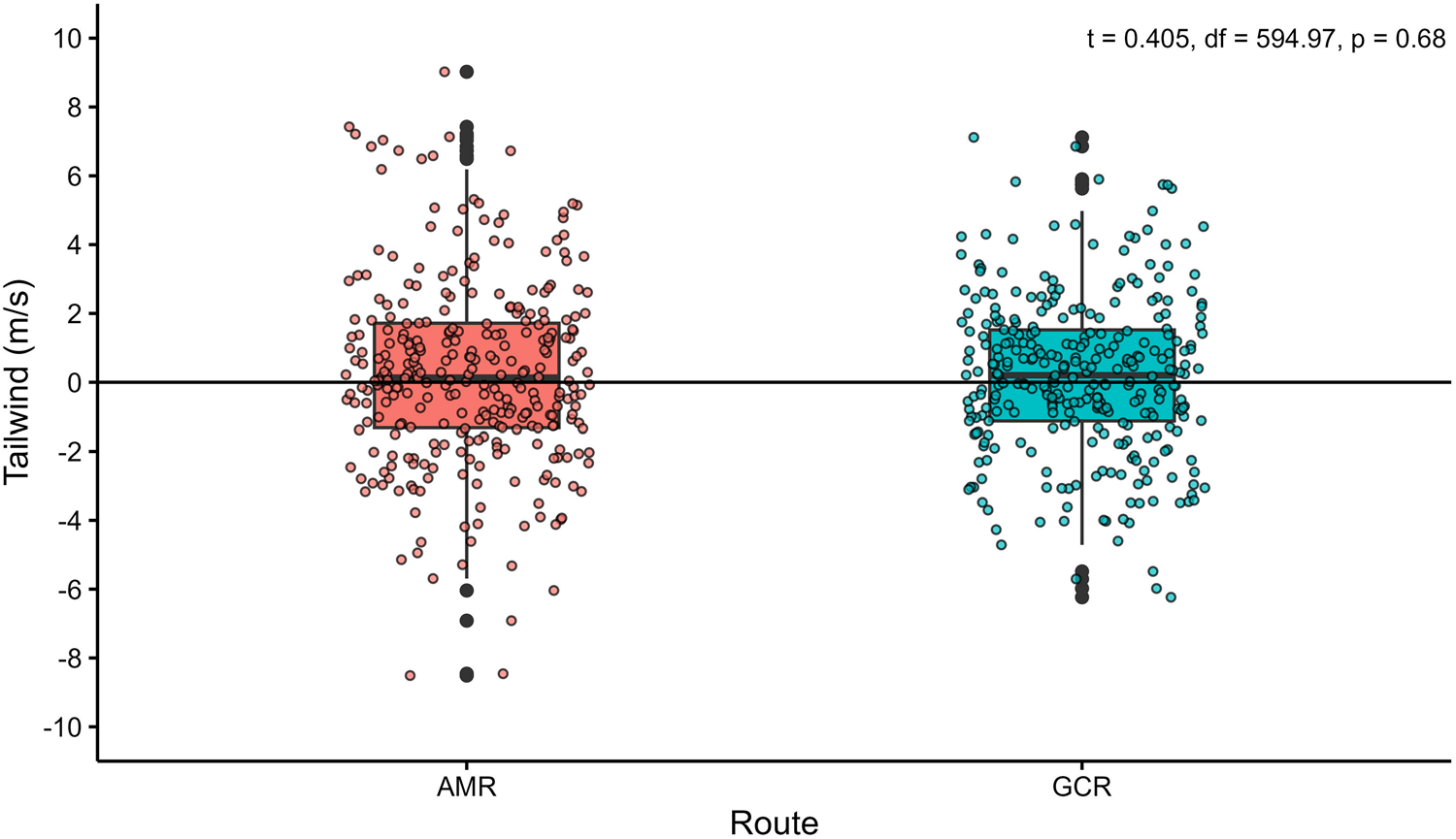
Box plot showing tailwind support for Montagu’s Harriers between the AMR and GCR (N = 307) segments

Due to the combination of supportive winds in some regions along the migration route and opposing winds in other regions, the overall average wind support was close to zero. This was true for autumn and spring migration, and also no difference was found in the wind support between the AMRs and the GCRs (Fig. 7).

## Discussion

### Detour migration by Montagu’s Harriers

By tagging Montagu’s Harriers with PTT and GPS-GSM tags in their wintering range in India, we set out to reveal the migration pattern of this declining bird of prey, adding novel information about migration routes within the understudied CAF [21]. We found that the tracked Montagu’s Harriers migrated via a long western detour route that was on average 27% longer than the shortest great circle route (GCR). This large detour occurred both in autumn and in spring. Extreme detours like this have previously been observed in a few species as a result of avoiding unfavourable habitats such as large sea crossings [58], unavailability of high-quality stopover sites [59] or to capture favourable winds to reduce the energetic costs of migratory journeys [7]. In the CAF, several raptor species are known to detour the Himalayas either to the west or east of the mountain range (see [60]), however empirical data on the exact geometry of the detour route and the extent of the detour was lacking.

Tracking studies of migrants in the CAF hitherto mainly focussed on species that undertake Trans Himalayan flights, because of the interest in (the physiology of) extreme high altitude migration [19, 21, 61]. Many of these species crossing the Himalayas in one season would take a detour in the other season, because of access to foraging habitats [62, 63] or as a result of their evolutionary history [21, 64]. The migration pattern in the Montagu’s Harrier is the sole example of detour migrations during both autumn and spring migration.

### Optimality of the detour

Based on Alerstam’s [12] maximum detour model, we calculated that the predicted maximum detour distance (1288 km) (just about) exceeded the observed detour distance (on average 1235 km). Thus, from this cost–benefit analysis, the detour made by the Montagu’s Harriers within the CAF seems optimal. Previous evaluations of the optimality of detours did not always come to this conclusion. For example, the detours of the Red-backed Shrike *Lanius collurio* (Tøttrup et al. [65]) and Short-toed Eagle *Circaetus gallicus* [58] were considered sub-optimal, which could be explained by the relatively short barrier distance (Mediterranean Sea, ~ 140 km). One of the reasons why the long detour made by the Montagu’s Harriers in the CAF could still be optimal is the fact that the barrier distance (the Himalayan plateau) was notably long (~ 700 km).

Alerstam’s [12] maximum detour model builds on the assumption that transport costs are higher during the barrier crossing than during the detour, as barriers are crossed by energy demanding active flapping flight whereas during the detour, the birds travel by energetically efficient soaring flight. This assumption might be true for facultative soaring migrants crossing barriers like water bodies [58, 66], but it might be a too simplified view for Montagu’s Harriers crossing a mountain range. Montagu’s Harriers are known to be flight generalists that alternate between flapping and soaring flight during migration [33]. We do not know how the ratio between flapping and soaring flight would vary with altitude and thus what the extra relative costs of transportation during the crossing of the Himalayas would be, but presumably this ratio is smaller than the threshold value of 2.84 that would make longer detour migration optimal. On the other hand, flight costs are generally higher at higher altitudes [67], which would make the costs of transport during the barrier crossing higher, irrespective of flight modes. Thus, the question whether the detour migration around a high-altitude range is optimal can only be answered if we would have a better understanding of flight behaviour and the energetic costs of the different flight modes at different altitudes.

### Is the Himalayan plateau too high for the harriers to cross?

Montagu’s Harriers circumvented the Himalayas by making a large detour in the CAF. If they had travelled along the shorter GCR, they would regularly have encountered high ground elevations between 4000 and 6000 m AMSL (for a distance of ~ 700 km). During the detour migration, the highest ground elevation the birds encountered was 4503 m. For the subset of the individuals we tracked with GPS-GSM tags, it was found that Montagu’s Harriers rarely occurred above flight altitudes of > 4000 m AMSL. Thus clearly, Montagu’s Harriers avoided the high elevations of the Himalayas by making the detour.

Birds generally can fly at high altitudes [19, 22, 68], but the challenging conditions of low air density, low oxygen pressure and freezing temperatures require specific physiological adaptations [67, 69]. Montagu’s Harriers and other medium-sized raptors might simply lack these adaptations and therefore avoid high altitudes. Although the record breaking observation of a Rüppell's Vulture *Gyps rueppelli* soaring at 11,278 m [70] shows that soaring birds can reach extreme altitudes using (extreme) thermal updrafts, soaring conditions generally are suboptimal at high altitudes because of the low density of the air [22]. Montagu’s Harriers are flight generalists that switch between flapping and soaring flights during migration depending on the availability of thermals [33, 71]. Theoretically, the low wing loading of Montagu’s Harriers should enable them to reach climbing rates similar to those of larger raptors such as Buzzards and Eagles [33]. However, previous studies show that Montagu’s Harriers are unable to achieve higher climbing rates possibly due to the V-shaped position of the wing during flight, reducing the effect of thermal updrafts for soaring flight [33]. Thus, a time minimization strategy by frequently switching to flapping flight to extend daily migration distances along with the lack of good soaring conditions makes high altitude travels unfeasible for migration, at least for the Montagu’s Harrier and possibly the other species of harriers using the CAF. The frequent switch to energy demanding flapping flights to compensate for lower cross-country speeds of soaring-gliding flight [72] and when soaring conditions deteriorate, results in particularly high metabolic rates at high altitudes [67]. Thus, a western detour migration route around the Himalayas in the CAF seems to be more adaptive for Montagu’s Harriers in order to avoid high altitudes of > 4000 m AMSL.

### The role of habitat and wind factors

Open natural ecosystems (ONEs) and croplands are considered to be the most important foraging habitats for Montagu’s Harriers [30, 73, 74]. These habitats were abundant along both the AMR and the GCR, although the AMR had proportionally more ONEs such as savanna grasslands, a preferred habitat of Montagu’s Harrier, compared to the GCR. From this perspective, the AMR and GCR both would offer plenty of foraging opportunities along the way. However, apart from the mountainous habitats including ice capped peaks and glaciers at high elevations, harriers would be faced with two additional stretches of unsuitable habitat along the GCR. These are the densely forested areas at the foothills of the Himalayas and central India, and the Taklamakan Desert (38.99°N, 82.81°E) north of the Himalayas. The Taklamakan Desert is a sandy expanse and is one of the major ecological barriers in the CAF that many bird species are known to circumvent [60]. Furthermore, along the AMR we also identified two prime stopover sites that are visited by the harriers during periods of maximum seasonal productivity. From this perspective, one could argue that the AMR generally provides more suitable foraging habitats all along the route than the GCR. Thus, the more favourable habitats along the AMR certainly facilitates the evolution of this detour.

Different soaring migrants are believed to make detour migrations to take advantage of favourable winds (i.e. [7]). Could this explain the detour made by the Montagu’s Harriers? The wind drift analysis showed that the daily progress of migrating harriers clearly was influenced by wind. However, generic wind patterns in this region do not always support the detour migration pattern in the harriers. For example, in autumn, only the start of the migration was supported by winds blowing in the direction of the migration route in the higher latitudes, resulting in a high proportion of drift, whereas in the latter stages, the winds were often opposing or blowing from the side, forcing the birds to frequently compensate or even overcompensate. In spring, the overall correlation between perpendicular movement and crosswind was non-significant, indicating a large component of compensation and overcompensation. Also, the overall (average) wind support was notably marginal, and this did not differ between the AMR and the GCR. Wind also did not seem to affect the active travel speeds of Montagu’s Harriers in spring which can be a time minimization strategy that is commonly exhibited by migrating birds during spring migration [75]. Finally, migration routes shaped by generic wind patterns typically result in loop migrations, with spring routes differing from autumn routes [4, 38, 76]. The case of Montagu’s Harriers in the CAF is strikingly different as the autumn and spring routes are alike. We thus conclude that the detour migration pattern of Montagu’s Harriers is not shaped by wind. This is also because Montagu’s Harriers are known to switch to flapping flight in conditions when winds are not favourable [33, 55]. In other words, generic wind patterns do not always dictate migration routes, despite its large effect on the speed and direction of the movement of flying birds. Factors such as altitudinal barriers might be more important in shaping migration routes.

## Conclusion

### Implications for the conservation of migrating birds within the Central Asian Flyway

Our tracking study has revealed that Montagu’s Harriers migrate via a fairly narrow corridor in the CAF constrained by the high elevations of the Himalayas towards the eastern side of the corridor. Although the amount of tracking studies in this part of the world is still limited, it seems that more raptors in the CAF migrate via this western circum-Himalayan corridor [77], highlighting the importance of protecting this flyway for the conservation of long distance migrating species. Loss or degradation of ONEs within this migration corridor might be detrimental to populations of Montagu’s Harriers and other migrants in the CAF. This is especially important given how often ONEs have received very little conservation attention [78]. The routes more to the west of the AMR seem unfavourable because it would further extend the already long migration detours these species undertake. Although currently, the observed detour seems to be energetically optimal in comparison to the theoretical maximal detour, a further shift of migration routes to the west as a result of unfavourable changes to the landscapes will make the detour sub-optimal due to the increased migration distances.

Little is known about where migratory birds make stopovers in the CAF, especially raptors [60]. We discovered two main stopovers for the Montagu’s Harriers, the Thar Desert in autumn and the floodplains of the Amu Darya river in spring. The autumn stopover in the Thar Desert seems to be a key stopover site for harriers in terms of regularity, the number of individuals making this stopover and the duration of this stopover. The Thar Desert receives the south-west monsoon from June to September, resulting in a flush of productivity for the next couple of months [79], making the desert suitable for a wide variety of prey such as grasshoppers, reptiles and birds [40]. This is exactly the moment Montagu’s Harriers are making their stopovers in this region. The floodplains of the Amu Darya river were the most important stopover site in spring, although only used by a minority of the birds and for relatively short times. This site also experienced a flush in productivity in the exact time period it was used by the harriers. This resembles the use of the northern part of the Sahel by Montagu’s Harriers in the Afro-Palearctic migration system [38]. The Sahel is also fed by summer rains, boosting productivity from which the harrier’s profit in early winter, just after they have crossed the barrier of the Sahara Desert [80]. Later tracking studies on other species of migrants revealed the general importance of this region for spring stopovers [76, 81, 82]. Montagu’s Harriers are just one of many species exploiting these temporal flushes in productivity, in the Thar Desert and the floodplains of the Amur Darya river. But, there could be other areas along the flyway that may be important in the CAF that needs to be identified. This potentially makes these regions, areas of great conservation concern, and therefore important destinations for future research on the stopover ecology of Montagu’s Harriers and other migrating birds in the understudied CAF.

## Supplementary Information

Additional file 1.

## Data Availability

The tracking data for this study is available in Movebank Digital Data Repository (movebank.org) and can be accessed upon requesting the authors.

## Abbreviations

CAF: Central Asian Flyway
NDVI: Normalized difference vegetation index
AMR: Actual migration route
GCR: Great circle route
LCLU: Land cover land use
AMSL: Above mean sea level
ONE: Open natural ecosystems
