# Supplementary material for: Detour migration to circumvent the Himalayas in the Montagu’s Harrier Circus pygargus
Source: Mov Ecol. 2025 Jun 10;13:40. doi: 10.1186/s40462-025-00568-z (PMC12153142; doi:10.1186/s40462-025-00568-z)
Supplement: Supplementary file 1 — Additional file 1. [file 40462_2025_568_MOESM1_ESM.docx]

**Table S1.** The table shows data for the 40 migration tracks of the 9 tagged Montagu’s Harriers tracked in 2017-2021.

| %Detour | 18.54 | 20.09 | 21.35 | 29.47 | 19.12 | 23.37 | 32.92 | 26.96 | 14.23 | 11.23 | 21.94 | 22.91 | 23.53 | 23.12 | 20.66 | 18.92 | 25.99 | 24.10 | 26.26 | 29.05 | 23.06 | 29.21 | 21.18 | 20.82 | 23.36 | 26.64 | 24.63 | 23.43 | 28.73 | 28.26 | 18.69 | 29.63 | 43.45 | 58.44 | 34.25 | 32.14 | 26.27 | 24.11 | 37.38 | 37.12 |
| --- | --- | --- | --- | --- | --- | --- | --- | --- | --- | --- | --- | --- | --- | --- | --- | --- | --- | --- | --- | --- | --- | --- | --- | --- | --- | --- | --- | --- | --- | --- | --- | --- | --- | --- | --- | --- | --- | --- | --- | --- |
| Detour distance | 741.48 | 852.28 | 1163.9 | 1023.6 | 799.58 | 962.3 | 1198.5 | 898.56 | 752.57 | 453.5 | 1229 | 1305.1 | 792.45 | 619.24 | 862.26 | 797.55 | 1539.1 | 1391 | 1241.2 | 1009 | 754.22 | 817.5 | 918.5 | 903.33 | 1047.2 | 1211.4 | 1430.5 | 1344.1 | 1404.2 | 1371.4 | 786.5 | 1386.7 | 1997.4 | 3800.3 | 1960.9 | 1482 | 1560 | 1392.9 | 2164.1 | 2438.7 |
| GCR Distance | 3257.4 | 3389.2 | 4286.7 | 2449.3 | 3381.7 | 3156.2 | 2442.1 | 2434.4 | 4534.7 | 3584 | 4372.9 | 4391.4 | 2575.2 | 2059.1 | 3311.3 | 3416.8 | 4382.8 | 4380.2 | 3485.3 | 2464.6 | 2516.1 | 1981.1 | 3417.3 | 3435.3 | 3435.3 | 3336.1 | 4377.3 | 4393.8 | 3483.1 | 3481.8 | 3422.1 | 3293.2 | 2599.4 | 2702.2 | 3764.8 | 3128.9 | 4378.2 | 4383.3 | 3626.2 | 4131.8 |
| AMR Distance | 3998.9 | 4241.4 | 5450.5 | 3472.9 | 4181.3 | 4118.5 | 3640.6 | 3332.9 | 5287.3 | 4037.5 | 5601.9 | 5696.5 | 3367.6 | 2678.4 | 4173.5 | 4214.4 | 5921.9 | 5771.2 | 4726.5 | 3473.5 | 3270.4 | 2798.6 | 4335.8 | 4338.6 | 4482.5 | 4547.5 | 5807.8 | 5738 | 4887.3 | 4853.2 | 4208.6 | 4679.9 | 4596.8 | 6502.4 | 5725.8 | 4610.9 | 5938.3 | 5776.2 | 5790.3 | 6570.5 |
| Stopover days | 0 | 16 | 0 | 4 | 4 | 50 | 0 | 15 | 5 | 8 | 0 | 9 | 7 | 10 | 6 | 43 | 0 | 10 | 0 | 7 | 5 | 0 | 11 | 12 | 7 | 52 | 0 | 28 | 0 | 71 | 15 | 0 | 7 | 69 | 15 | 20 | 0 | 22 | 12 | 3 |
| No. of stopovers | 0 | 2 | 0 | 0 | 1 | 3 | 0 | 3 | 1 | 1 | 0 | 1 | 1 | 1 | 1 | 2 | 0 | 2 | 0 | 2 | 1 | 0 | 3 | 2 | 1 | 2 | 0 | 2 | 0 | 3 | 2 | 0 | 1 | 6 | 4 | 1 | 0 | 1 | 2 | 1 |
| Active travel speed | 166.61 | 176.72 | 194.66 | 315.71 | 199.10 | 187.20 | 158.28 | 302.99 | 251.77 | 269.16 | 215.45 | 167.54 | 280.63 | 178.55 | 231.86 | 168.57 | 236.87 | 164.89 | 205.5 | 248.10 | 172.12 | 174.91 | 197.08 | 228.34 | 249.02 | 239.34 | 232.31 | 204.92 | 222.15 | 255.43 | 263.03 | 150.96 | 208.94 | 224.22 | 357.85 | 230.54 | 228.39 | 175.03 | 160.84 | 193.24 |
| Active travel days | 24 | 24 | 28 | 11 | 21 | 22 | 23 | 11 | 21 | 15 | 26 | 34 | 12 | 15 | 18 | 25 | 25 | 35 | 23 | 14 | 19 | 16 | 22 | 19 | 18 | 19 | 25 | 28 | 22 | 19 | 16 | 31 | 22 | 29 | 16 | 20 | 26 | 33 | 36 | 34 |
| Travel speed | 166.61 | 106.03 | 194.66 | 315.71 | 167.25 | 57.20 | 158.28 | 128.189 | 203.35 | 175.54 | 215.45 | 132.47 | 177.24 | 107.13 | 173.89 | 61.97 | 236.87 | 128.24 | 205.5 | 165.40 | 136.26 | 174.91 | 131.38 | 139.95 | 179.29 | 64.04 | 232.31 | 102.46 | 222.15 | 53.92 | 135.76 | 150.96 | 158.51 | 66.35 | 184.70 | 115.27 | 228.39 | 105.02 | 120.63 | 177.58 |
| Travel Duration | 24 | 40 | 28 | 11 | 25 | 72 | 23 | 26 | 26 | 23 | 26 | 43 | 19 | 25 | 24 | 68 | 25 | 45 | 23 | 21 | 24 | 16 | 33 | 31 | 25 | 71 | 25 | 56 | 22 | 90 | 31 | 31 | 29 | 98 | 31 | 40 | 26 | 55 | 48 | 37 |
| Arrival day | 137 | 259 | 112 | 253 | 115 | 258 | 104 | 262 | 112 | 263 | 114 | 276 | 114 | 273 | 106 | 284 | 114 | 280 | 104 | 260 | 107 | 255 | 113 | 257 | 112 | 299 | 115 | 283 | 108 | 317 | 255 | 113 | 127 | 279 | 123 | 275 | 120 | 284 | 320 | 107 |
| Arrival date | 17-05-2017 | 16-09-2017 | 22-04-2017 | 10-09-2017 | 25-04-2018 | 15-09-2018 | 14-04-2018 | 19-09-2018 | 22-04-2018 | 20-09-2018 | 24-04-2018 | 03-10-2018 | 24-04-2018 | 30-09-2018 | 16-04-2019 | 11-10-2019 | 24-04-2019 | 07-10-2019 | 14-04-2019 | 17-09-2019 | 17-04-2019 | 12-09-2019 | 22-04-2020 | 13-09-2020 | 21-04-2020 | 25-10-2020 | 24-04-2020 | 09-10-2020 | 18-04-2021 | 13-11-2021 | 12-09-2021 | 23-04-2021 | 07-05-2021 | 06-10-2021 | 03-05-2021 | 02-10-2021 | 30-04-2021 | 11-10-2021 | 16-11-2021 | 17-04-2021 |
| Departure day | 113 | 218 | 84 | 242 | 91 | 184 | 81 | 236 | 86 | 240 | 88 | 234 | 95 | 248 | 82 | 216 | 90 | 236 | 81 | 239 | 83 | 239 | 80 | 226 | 87 | 227 | 91 | 228 | 86 | 227 | 224 | 82 | 98 | 181 | 92 | 235 | 94 | 229 | 241 | 80 |
| Departure date | 23-04-2017 | 06-08-2017 | 25-03-2017 | 30-08-2017 | 01-04-2018 | 03-07-2018 | 22-03-2018 | 24-08-2018 | 27-03-2018 | 28-08-2018 | 29-03-2018 | 22-08-2018 | 05-04-2018 | 05-09-2018 | 23-03-2019 | 04-08-2019 | 31-03-2019 | 24-08-2019 | 22-03-2019 | 27-08-2019 | 24-03-2019 | 27-08-2019 | 20-03-2020 | 13-08-2020 | 27-03-2020 | 14-08-2020 | 31-03-2020 | 15-08-2020 | 27-03-2021 | 15-08-2021 | 12-08-2021 | 23-03-2021 | 08-04-2021 | 30-06-2021 | 02-04-2021 | 23-08-2021 | 04-04-2021 | 17-08-2021 | 29-08-2021 | 21-03-2021 |
| Season | Spring | Autumn | Spring | Autumn | Spring | Autumn | Spring | Autumn | Spring | Autumn | Spring | Autumn | Spring | Autumn | Spring | Autumn | Spring | Autumn | Spring | Autumn | Spring | Autumn | Spring | Autumn | Spring | Autumn | Spring | Autumn | Spring | Autumn | Autumn | Spring | Spring | Autumn | Spring | Autumn | Spring | Autumn | Autumn | Spring |
| Year | 2017 | 2017 | 2017 | 2017 | 2018 | 2018 | 2018 | 2018 | 2018 | 2018 | 2018 | 2018 | 2018 | 2018 | 2019 | 2019 | 2019 | 2019 | 2019 | 2019 | 2019 | 2019 | 2020 | 2020 | 2020 | 2020 | 2020 | 2020 | 2021 | 2021 | 2021 | 2021 | 2021 | 2021 | 2021 | 2021 | 2021 | 2021 | 2021 | 2021 |
|  |  |  |  |  |  |  |  |  |  |  |  |  |  |  |  |  |  |  |  |  |  |  |  |  |  |  |  |  |  |  |  |  |  |  |  |  |  |  |  |  |
| Logger | PTT | PTT | PTT | PTT | PTT | PTT | PTT | PTT | PTT | PTT | PTT | PTT | PTT | PTT | PTT | PTT | PTT | PTT | PTT | PTT | PTT | PTT | PTT | PTT | GSM | GSM | PTT | PTT | GSM | GSM | PTT | PTT | GSM | GSM | GSM | GSM | PTT | PTT | GSM | GSM |
| Bird ID | Gange | Gange | Paruthi | Paruthi | Gange | Gange | Paruthi | Paruthi | Deo | Deo | Nellai | Nellai | Rupeli | Rupeli | Gange | Gange | Nellai | Nellai | Paruthi | Paruthi | Rupeli | Rupeli | Gange | Gange | Dewani | Dewani | Nellai | Nellai | Dewani | Dewani | Gange | Gange | Hira | Hira | Mothiya | Mothiya | Nellai | Nellai | Rangaa | Rangaa |

**Table S2.** Combined ESA CCI land cover classes to the 8 thematic classes used for the analysis. Each thematic class was given a new unique ID for the analysis.

| ESA CCI Class ID | Combined class | New Unique ID |
| --- | --- | --- |
| 10, 20 | Cropland | 1 |
| 30 | Cropland mosaic | 2 |
| 12, 50, 60, 61, 62, 70, 71, 72, 80, 81, 82, 90, 100 | Forest | 3 |
| 11, 40, 110, 120, 121, 122, 130, 150, 151, 152, 153, 180 | Open Natural Ecosystem (ONE) | 4 |
| 190 | Urban area | 5 |
| 200, 201, 202 | Bare area | 6 |
| 210 | Waterbody | 7 |
| 220 | Permanent snow & ice | 8 |

**Table S3**. Test statistics of the difference in elevations between actual migration route (AMR) and great circle route (GCR) across seasons (autumn and spring) and across 10° latitudinal ranges. Values in bold indicate significant differences (*P* < 0.05)

| SRTM elevation | group | N | statistic | *P* |
| --- | --- | --- | --- | --- |
| Route | AMR, GCR | 17551, 1433 | 9220128 | **<0.001** |
| AMR Season | Autumn, Spring | 7986, 9565 | 35599857 | **<0.001** |
| GCR Season | Autumn, Spring | 664, 771 | 423445 | 0.32 |
| Lat_8_18-Season | Autumn, Spring | 890, 741 | 413348 | **<0.001** |
| Lat_8_18-Route | AMR, GCR | 1480, 151 | 146966 | **<0.001** |
| Lat_18_28-Season | Autumn, Spring | 2955, 2523 | 2764721 | **<0.001** |
| Lat_18_28-Route | AMR, GCR | 5086, 392 | 810193 | **<0.001** |
| Lat_28_38-Season | Autumn, Spring | 1703, 3352 | 2569482 | **<0.001** |
| Lat_28_38-Route | AMR, GCR | 4598, 457 | 534748 | **<0.001** |
| Lat_38_48-Season | Autumn, Spring | 3054, 3341 | 6366882 | **<0.001** |
| Lat_38_48-Route | AMR, GCR | 5977, 418 | 546340 | **<0.001** |
| Lat_48_58-Season | Autumn, Spring | 47, 378 | 10928 | **0.01** |
| Lat_48_58-Route | AMR, GCR | 410, 15 | 2419 | 0.16 |

**Table S4.** Test showing differences in proportion of locations over the 8 land cover types across AMR and GCR. “Combined” indicates all locations along AMR and GCR without splitting the migration seasons. Significant results (*P* < 0.05) have been shown in bold.

| Season | Land cover | ̜χ^2^ | Lower CI | Upper CI | df | *P* |
| --- | --- | --- | --- | --- | --- | --- |
| Combined | Cropland | 68.152 | -0.0671 | -0.0407 | 1 | **<0.01** |
|  | Cropland mosaic | 0.018582 | -0.0048 | 0.0042 | 1 | 0.89 |
|  | Forest | 496.82 | -0.0576 | -0.0437 | 1 | **<0.01** |
|  | ONE | 817.79 | 0.1840 | 0.2087 | 1 | **<0.01** |
|  | Urban area | 225.31 | -0.0118 | -0.0065 | 1 | **<0.01** |
|  | Bare area | 145.1 | -0.0622 | -0.0426 | 1 | **<0.01** |
|  | Waterbody | 6.742 | -0.0076 | -0.0006 | 1 | 0.009 |
|  | Permanent snow & ice | 1576.3 | -0.0283 | -0.0203 | 1 | **<0.01** |
| Autumn | Cropland | 20.674 | -0.0626 | -0.0244 | 1 | **<0.01** |
|  | Cropland mosaic | 0.986 | -0.0028 | 0.0093 | 1 | 0.32 |
|  | Forest | 202.69 | -0.0528 | -0.0341 | 1 | **<0.01** |
|  | ONE | 538.15 | 0.2129 | 0.2486 | 1 | **<0.01** |
|  | Urban area | 95.185 | -0.0112 | -0.0042 | 1 | **<0.01** |
|  | Bare area | 635.15 | -0.1346 | -0.1060 | 1 | **<0.01** |
|  | Waterbody | 1.096 | -0.0021 | -0.0077 | 1 | 0.29 |
|  | Permanent snow & ice | 654.93 | -0.0272 | -0.0162 | 1 | **<0.01** |
| Spring | Cropland | 10.843 | -0.0510 | -0.0126 | 1 | **<0.01** |
|  | Cropland mosaic | 0.038 | -0.0080 | 0.00651 |  | 0.84 |
|  | Forest | 249.05 | -0.0704 | -0.0484 | 1 | **<0.01** |
|  | ONE | 541.84 | 0.2131 | 0.2494 | 1 | **<0.01** |
|  | Urban area | 117.2 | -0.0155 | -0.0069 | 1 | **<0.01** |
|  | Bare area | 8.895 | 0.0085 | 0.0375 | 1 | **<0.01** |
|  | Waterbody | 22.794 | -0.0154 | -0.0045 | 1 | **<0.01** |
|  | Permanent snow & ice | 936.18 | -0.0384 | -0.0252 | 1 | **<0.01** |

**Table S5.** Linear regressions between rate of forward movement (km/day) and forward wind component (m/s) and between rate of perpendicular movement (km/day) and perpendicular movement component (m/s). Significant results (*P* < 0.05) have been shown as bold numbers in the table. Significance level of the difference between the slopes according to the 95% confidence interval are also shown (* *P*  > 0.05, n.s.: not significant)

| Season | N | Forward movement | | | Perpendicular movement | | | Test between slopes | Ratio b/w slopes (perp/fwd) | |
| --- | --- | --- | --- | --- | --- | --- | --- | --- | --- | --- |
|  |  | Slope | 95% confidence | Intercept | Slope | 95% confidence | Intercept |  |  |  |
| All | 338 | **23.17** 16.50-29.85 223.55 | | | **13.23** 5.49-20.97 -13.40 | | | ***** | 0.57 |  |
| Autumn | 169 | **29.4** 18.98-40.84 181.810 | | | **24.68** 13.36-35.99 -25.85 | | | n.s | 0.83 |  |
| Spring | 169 | **24.69** 16.07-33.31 260.47 | | | 5.05 -5.89-16.00 -11.54 | | | ***** | 0.20 |  |

**Table S6.** Percentage of drift, compensation and overcompensation segments between the three latitudes during autumn and spring migrations of Montagu’s harriers. Significant results (*P* < 0.05) has been shown in bold

| Migration season | Latitude | Segments (%) | | | N | χ2 | *P* |
| --- | --- | --- | --- | --- | --- | --- | --- |
|  |  | Drift | Compensation | Overcompensation |  |  |  |
| Autumn | >40°N | 62.22 | 15.55 | 22.22 | 169 | 20.064 | **<0.01** |
|  | 40°N -30°N | 31.42 | 22.85 | 45.71 |  |  |  |
|  | <30°N | 34.83 | 42.69 | 22.47 |  |  |  |
| Spring | >40°N | 46.1 | 3.84 | 50 | 169 | 21.041 | **<0.01** |
|  | 40°N -30°N | 63.26 | 20.4 | 16.32 |  |  |  |
|  | <30°N | 33.82 | 22.05 | 44.11 |  |  |  |
